# Acute pancreatitis risk after kidney transplantation: Propensity score matching analysis of a national cohort

**DOI:** 10.1371/journal.pone.0222169

**Published:** 2019-09-11

**Authors:** Ya-Wen Chuang, Shih-Ting Huang, Tung-Min Yu, Chi-Yuan Li, Mu-Chi Chung, Cheng-Li Lin, Chi-Sen Chang, Ming-Ju Wu, Chia-Hung Kao

**Affiliations:** 1 Division of Nephrology, Taichung Veterans General Hospital, Taichung, Taiwan; 2 College of Medicine, China Medical University, Taichung, Taiwan; 3 Graduate Institute of Public Health, China Medical University, Taichung, Taiwan; 4 Graduate Institute of Biomedical Sciences and School of Medicine, College of Medicine, China Medical University, Taichung, Taiwan; 5 Office for Health Data, China Medical University Hospital, Taichung, Taiwan; 6 Division of Gastroenterology, Department of Internal Medicine, Taichung Veterans General Hospital, Taichung, Taiwan; 7 Department of Nuclear Medicine and PET Center, and Center of Augmented Intelligence in Healthcare, China Medical University Hospital, Taichung, Taiwan; 8 Department of Bioinformatics and Medical Engineering, Asia University, Taichung, Taiwan; Imperial College Healthcare NHS Trust, UNITED KINGDOM

## Abstract

**Purpose:**

Data for elucidating post-kidney transplantation (KT) acute pancreatitis (AP) risk are limited and no large-scale cohort study has investigated the impact of AP after KT.

**Method:**

Data from Taiwan National Health Insurance (NHI) Research Database (NHIRD) were calculated through the method of propensity score matching to compare the pancreatitis risk in patients with and without KT.

**Results:**

The overall pancreatitis incidence rates were 1.71 and 0.61 per 1,000 person-years in the KT and non-KT groups, respectively and corresponding adjusted HR (aHR [95% CI]) for pancreatitis was 2.48 (1.51–4.09) in the KT group. In the multivariable model, AP risk was higher in transplant patients with alcohol-related illnesses (aHR: 3.78, 95% CI: 1.32–10.8), gall stone disease (aHR: 3.53, 95% CI: 1.48–8.44), or past history of pancreatitis (aHR: 10.3, 95% CI: 5.08–20.8). Of note, recurrent AP risk was significantly higher in the KT group (aHR: 8.19, 95% CI: 2.89–23.2). Patients with post-KT AP demonstrated shorter patient and allograft survival than did those without (both *P* < 0.001, respectively).

**Conclusion:**

In conclusion, KT recipients are very likely to be associated with AP. Moreover, their inferior outcomes are strongly associated with post-KT AP.

## Introduction

Pancreatitis, a debilitating inflammatory condition of the pancreatic body, is often associated with substantial mortality rates in patients [1, 2]. In the general population, the estimated incidence rate is 0.04–0.45 per 1000 person-years [3, 4], with fatality noted in 2%–5% of the patients [1, 2]. Pancreatitis has risk factors, including gall stone disease, heavy alcohol consumption, hypertriglyceridemia, viral hepatitis, and diabetes mellitus [5, 6]. Nevertheless, regarding to the issue for pancreatitis management, it has been almost nonspecific, including supportive therapy alone.

Accumulating evidence suggests that chronic renal failure is significantly associated with acute pancreatitis (AP). AP may also be associated with higher mortality rates in patients with end-stage renal disease (ESRD) than that in the general population. In a study [7], the crude AP incidence rate in ESRD patients was 5.17 (95% confidence intervals [CI] 4.90–5.44) per 1000 person-years, with an in-hospital mortality rate of approximately 8.1%. Thus, ESRD increased the AP risk several fold; nevertheless, AP could be very likely overlooked in patients with ESRD. The development of serious complications in AP could be multifactorial in patients with ESRD; however, the uremic toxin is suggested to play an important role within it [8].

Compared to those receiving dialysis, kidney transplantation (KT) has favorable outcomes in patients with ESRD. However, even after transplantation, these renal kidney recipients are suggested to may be likely associated with a high pancreatitis AP risk as well. Before transplantation, substantial recipients likely acquire several comorbidities, such as diabetes mellitus, hyperlipidemia, viral infection, and hyperparathyroidism [9]; nevertheless, all of which remain even after transplantation. Moreover, most transplant recipients would have immunosuppression lifelong. Studies have suggested that immunosuppressants (ISs), including corticosteroid, cyclosporine, and azathioprine, likely cause pancreatic duct injury. Thus, these immunosuppressant medications in kidney recipients could become a major concern with regard to AP [10].

Data for elucidating post-KT AP risk are limited. Thus far, no large-scale cohort study has investigated the impact of AP after KT. Therefore, the study determined the association KT and AP by using data from a national population-based cohort with a long follow-up duration; furthermore, we identified the possible factors contributing to AP in kidney recipients.

## Methods

### Data source

We conducted this national population-based retrospective cohort study by using insurance data from Taiwan National Health Insurance (NHI) Research Database (NHIRD). The NHI program was established in March 1995. In 2015, its enrollment rate had exceeded 99% of all 23.7 million residents of Taiwan. The details of the NHI program and the NHIRD have been reported previously [11, 12].

### Ethics statement

The NHIRD encrypts patient personal information to protect privacy and provides researchers with anonymous identification numbers associated with relevant claims information, including sex, date of birth, medical services received, and prescriptions. Therefore, patient consent is not required to access the NHIRD. This study was approved to fulfill the condition for exemption by the Institutional Review Board (IRB) of China Medical University (CMUH104-REC2-115-CR3). The IRB also specifically waived the consent requirement.

### Patients

We included KT patients aged ≥18 years between 1998 and 2010 (ICD-9-CM code V42.0, 996.81) in the KT group. The date of the first hospitalization for KT was considered to be the index date. The exclusion criteria were age < 18 years; pancreas transplantation or missing data regarding date of birth or sex. For comparison, each KT patient was propensity score-matched with an individual without KT. The propensity scores were calculated through logistic regression to estimate the KT status and the baseline variables, including sex, age, index date year, alcohol-related illnesses, gall stone disease; hepatitis C virus (HCV), hepatitis B virus (HBV), and cytomegalovirus (CMV) infections; polycystic kidney disease, hyperlipidemia, and diabetes mellitus [13].

### Outcomes, medications, and comorbidities

Pancreatitis (ICD-9-CM: 577), including AP (ICD-9-CM: 577.0) and chronic pancreatitis (ICD-9-CM: 577.1), was defined as the endpoint of this study. All included patients were followed from the index date until the occurrence of the endpoint, withdrawal from the NHI program, or the end of 2011. We considered the following medications for KT treatment: steroids, tacrolimus, cyclosporin, sirolimus, mycophenolate mofetil, certican, and thymoglobulin. The potential comorbidities included alcohol-related illnesses, gall stone disease; HCV, HBV, and CMV infections; diabetes mellitus, polycystic kidney disease, hyperlipidemia, past history of pancreatitis, peritoneal dialysis, and hemodialysis.

### Statistical analysis

Standardized mean difference was used to compare the differences in the mean values and prevalence of continuous and categorical variables, respectively, between the KT and non-KT groups. A standardized mean difference of ≤ 0.10 indicates a negligible difference between the two groups. Cumulative incidence rates of AP in the KT and non-KT groups and the patient and allograft survival in AP and non-AP patients were explored using the Kaplan–Meier method. The intergroup differences were determined using log-rank tests. The incidence rates of pancreatitis, stratified by sex, age, and comorbidities, were calculated for both the KT and non-KT groups. Univariable and multivariable Cox proportional hazards models were used to estimate the hazard ratios (HRs) and their 95% CIs for KT-associated pancreatitis, AP, and chronic pancreatitis compared with the non-KT group. We examined the proportional hazard model assumption using a test of scaled Schoenfeld residuals. Results showed that there was no significant relationship between Schoenfeld residuals for kidney transplantation and follow-up time (p-value = 0.23, 0.41, 0.63, respectively) in the model evaluating the acute pancreatitis, chronic pancreatitis, and pancreatitis risk. Variables found to be statistically significant in the univariable model were further included in the multivariable model. Furthermore, we analyzed the risk of variables contributing to AP among the KT patients using the Cox model. All data analyses were performed using SAS version 9.4 (SAS Institute, Inc., Cary, NC, USA). The significance level was set at *P* < 0.05, and all tests were two-tailed.

## Results

Table 1 lists the demographic data and comorbidities for the KT (n = 5236) and non-KT (n = 5236) groups. Approximately 53% of the KT patients were men. The mean ages in the KT and non-KT group were 45.6 (±11.5 y) and 42.5 (±16.0 y), respectively. Compared with the non-KT group, more KT patients had comorbidities, including HCV infection (5.63% vs 0.88%), HBV infection (8.59% vs 3.38%), polycystic kidney disease (2.44% vs 0.04%), hyperlipidemia (31.8% vs 1.4%), and diabetes (14.2% vs 4.87%). The mean durations for pancreatitis follow-up were 6.57 ± 3.65 and 6.85 ± 3.74 years in the KT and non-KT group, respectively. Steroid use was more prevalent in the non-KT group than in the KT group. However, 65.6%, 55.2%, and 48.6% of KT patients were using mycophenolate mofetil, tacrolimus, and cyclosporin, respectively.

**Table 1:** Demographic characteristics and comorbidities in patients with and without kidney transplantation.

	Kidney transplantation		Standard mean difference
	No	Yes	
	(N = 5236)	(N = 5236)	
**Gender**			
Women	2600(49.7)	2468(47.1)	0.05
Men	2636(50.3)	2768(52.9)	0.05
**Age stratified**			
≤ 49	3692(70.5)	3246(62.0)	0.18
50–64	966(18.5)	1802(34.4)	0.37
≧65	578(11.0)	188(3.59)	0.29
Age, mean±SD	42.5±16.0	45.6±11.5	0.22
**Comorbidity**			
Alcohol-related illness	128(2.44)	110(2.10)	0.02
Gall stone	76(1.45)	133(2.54)	0.08
HCV	46(0.88)	295(5.63)	0.27
HBV	177(3.38)	450(8.59)	0.22
CMV	0(0.00)	9(0.17)	0.06
Polycystic kidney disease	2(0.04)	128(2.44)	0.22
Hyperlipidemia	598(11.4)	1667(31.8)	0.51
Diabetes	255(4.87)	743(14.2)	0.32
History of acute pancreatitis	28(0.53)	137(2.62)	<0.001
**Medications**			
Steroid	978(18.7)	209(3.99)	<0.001
Tacrolimus		2888(55.2)	
Cyclosporin		2545(48.6)	
Sirolimus		511(9.76)	
Mycophenolatemofetil		3591(65.6)	
Certican		3(0.06)	
Thymoglobuline		122(2.33)	

The overall pancreatitis incidence rates were 1.71 and 0.61 per 1,000 person-years in the KT and non-KT groups, respectively (Table 2). The corresponding adjusted HR (aHR [95% CI]) for pancreatitis was 2.48 (1.51–4.09) in the KT group, after adjustments for age, alcohol-related illness, gall stone, HCV, HBV, and diabetes. Moreover, compared with the non-KT group, KT patients exhibited 3.36 higher AP (95% CI: 1.88–6.01).

**Table 2:** Comparison of incidence and hazard ratio of pancreatitis between patients with and without kidney transplantation.

	Kidney transplantation							
	No			Yes				
	Event	PY	Rate^#^	Event	PY	Rate^#^	Crude HR (95% CI)	Adjusted HR^†^ (95% CI)
Acute Pancreatitis	15	35895	0.42	54	34440	1.57	3.77(2.13, 6.69)***	3.36(1.88, 6.01)***
Chronic Pancreatitis	9	35815	0.25	14	34577	0.40	1.62(0.70, 3.73)	1.50(0.64, 3.53)
Both	22	35865	0.61	59	34425	1.71	2.80(1.72, 4.58)***	2.48(1.51, 4.09)***

Rate^#^, incidence rate, per 1,000 person-years; Crude HR, relative hazard ratio; Variables found to be statistically significant in the univariable model were further included in the multivariable model.

^†^Adjusted for age, alcohol-related illness, gall stone, HCV, HBV, and diabetes.

***p<0.001.

In the KT group, the aHR (95% CI) for AP in women was 7.71 (2.68–22.2) (Table 3). The age-specific relative AP risk was significantly higher in KT patients aged ≤49 years compared with the non-KT group. The comorbidity-specific analyses showed that KT patients had a significantly higher AP risk than did those without KT and any comorbidity (aHR: 3.94, 95% CI: 1.83–8.48). Steroid, tacrolimus, cyclosporin, sirolimus, mycophenolate mofetil, and thymoglobulin were not significantly associated with AP development in the KT group (Table 4).

**Table 3:** Comparison of incidence and hazard ratio of acute pancreatitis between patients with and without kidney transplantation stratified by gender, age, and comorbidity.

	Kidney transplantation							
	No			Yes				
	Event	PY	Rate^#^	Event	PY	Rate^#^	Crude HR (95% CI)	Adjusted HR^†^ (95% CI)
**Gender**								
Women	4	17863	0.22	28	16566	1.69	7.57(2.65, 21.6)***	7.71(2.68, 22.2)***
Men	11	18031	0.61	26	17873	1.45	2.42(1.19, 4.89)*	1.97(0.95, 4.10)
P for interaction								0.32
**Stratify age**								
≤ 49	6	26219	0.23	33	23284	1.42	6.30(2.64, 15.0)***	5.24(2.15, 12.8)***
50–64	6	6178	0.97	20	10191	1.96	1.94(0.78, 4.85)	1.75(0.69, 4.46)
≧ 65	3	3498	0.86	1	964	1.04	1.11(0.12, 10.7)	1.77(0.13, 23.6)
P for interaction								0.07
**Comorbidity**^‡^								
No	9	30201	0.30	24	20202	1.19	4.04(1.88, 8.70)***	3.94(1.83, 8.48)***
Yes	6	5693	1.05	30	14238	2.11	2.01(0.83, 4.82)	2.39(0.96, 5.96)
P for interaction								0.24

Rate^#^, incidence rate, per 1,000 person-years; Crude HR, relative hazard ratio; Variables found to be statistically significant in the univariable model were further included in the multivariable model.

^†^Adjusted for age, alcohol-related illness, gall stone, HCV, HBV, and diabetes.

^**‡**^ Patients with any one of the comorbidities alcohol-related illness, gall stone, HCV, HBV, CMV, polycystic kidney disease, hyperlipidemia, and diabetes were classified as the comorbidity group

*p<0.05,

***p<0.001.

**Table 4:** Incidence rates and hazard ratios of acute pancreatitis among kidney transplantation patients stratified by treatment.

Variables	N	Event	Person-years	Rate^#^	Crude HR (95% CI)	Adjusted HR^†^ (95% CI)
**Steroid**						
No	5027	52	33007	1.58	1(Reference)	1(Reference)
Yes	209	2	1433	1.40	0.89(0.22, 3.64)	0.83(0.19, 3.65)
**Tacrolimus**						
No	2348	28	18747	1.49	1(Reference)	1(Reference)
Yes	2888	26	15692	1.66	1.05(0.61, 1.81)	1.01(0.41, 2.47)
**Cyclosporin**						
No	2691	25	14627	1.71	1(Reference)	1(Reference)
Yes	2545	29	19812	1.46	0.90(0.52, 1.56)	0.86(0.36, 2.03)
**Sirolimus**						
No	4725	50	31633	1.58	1(Reference)	1(Reference)
Yes	511	4	2807	1.43	0.88(0.32, 2.46)	0.80(0.28, 2.29)
**Mycophenolatemofetil**						
No	1645	21	12104	1.73	1(Reference)	1(Reference)
Yes	3591	33	22335	1.48	0.83(0.48, 1.44)	0.71(0.39, 1.29)
**Certican**						
No	5233	54	34456	1.57	1(Reference)	1(Reference)
Yes	3	0	3	0.00	-	-
**Thymoglobulin**						
No	5114	53	33856	1.57	1(Reference)	1(Reference)
Yes	122	1	583	1.71	1.05(0.15, 7.57)	1.15(0.16, 8.39)

Rate^#^, incidence rate, per 1,000 person-years; Crude HR, relative hazard ratio; Variables found to be statistically significant in the univariable model were further included in the multivariable model.

^†^Adjusted for alcohol-related illness, gall stone, HCV, and diabetes.

In the multivariable model, among the KT patients, AP risk was higher in patients with alcohol-related illnesses (aHR: 3.85, 95% CI: 1.36–10.9), gall stone disease (aHR: 3.43, 95% CI: 1.45–8.14), or past history of pancreatitis (aHR: 9.94, 95% CI: 4.98–19.8; S1 Table). Compared with the non-KT group, recurrent AP risk was significantly higher in the KT group (aHR: 9.77, 95% CI: 3.33–28.7; S2 Table).

**Fig 1:**
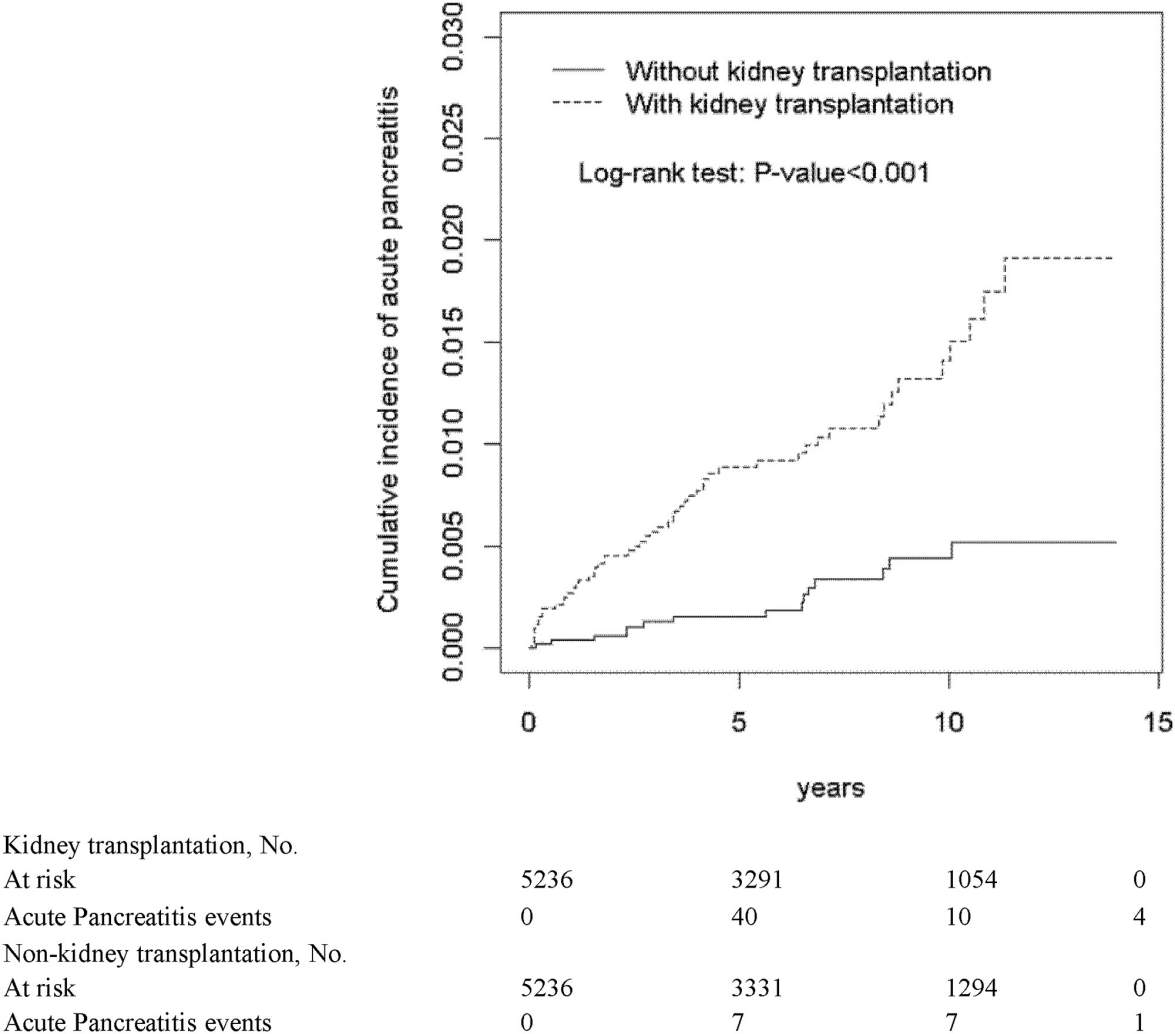
Cummulative incidence of acute pancreatitis between individuals with and without kidney transplantation.

**Fig 2:**
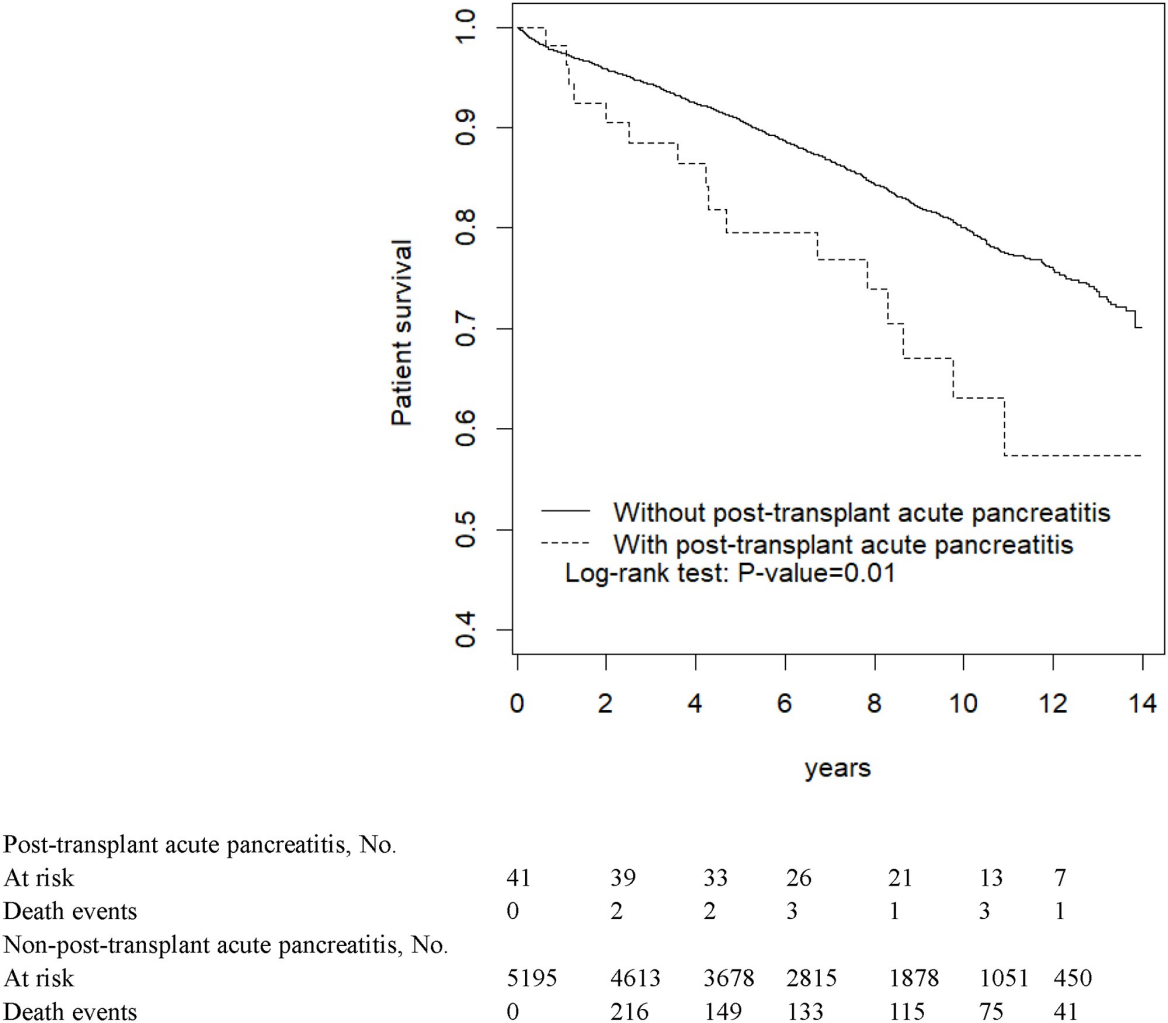
Survival of kidney transplant patients with and without acute pancreatitis.

**Fig 3:**
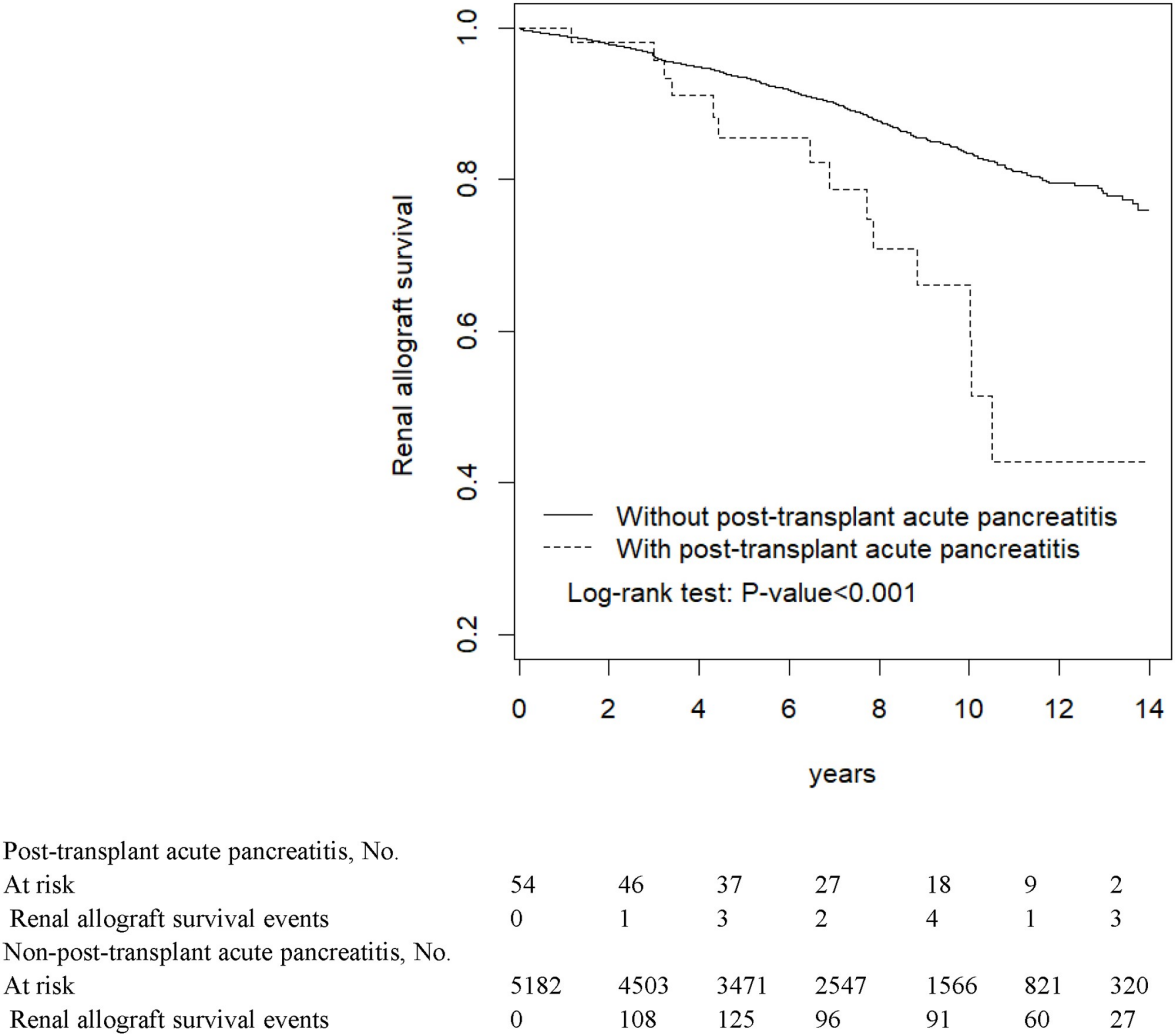
Survival of kidney allograft in patients with and without acute pancreatitis.

Fig 1 reveals that the KT group had a significantly higher cumulative proportion of AP patients than did the non-KT group (*P* < 0.001). Patients with post-KT AP demonstrated shorter patient and allograft survival than did those without (both *P* < 0.001; Figs 2 and 3, respectively).

## Discussion

This was a large-scale study with a long observational period. Our findings indicated that KT is significantly associated with up to 3.5-fold higher AP risk compared with the general population. Moreover, compared with the general population, post-KT AP risk was significantly higher in kidney recipients who were female or aged <50 years. Notably, those without any traditional factors, such as alcohol consumption, gall bladder stone disease, diabetes mellitus, hyperlipidemia, and viral hepatitis, had a 4.33-fold increased pancreatitis risk. ISs may be involved in AP development after KT. Thus, the patients’ IS medications were further analyzed; however, ISs were found to unlikely be associated with post-KT AP risk in the present study.

In most critical AP patients, supportive therapy through fluid infusion rather than oral intake is routinely applied in such situation. Even after AP subsided, the IS of these patients could be considered a possible cause of AP so that the IS would be attenuated to the minimal level as low as possible in the long run. Eventually, mild immunosuppressant may result in the consequent allograft rejection. In this study, compared with KT patients without pancreatitis, post-KT AP patients demonstrated a shorter patient and allograft survival. Studies have reported a considerably high mortality after a KT recipient develops AP [9]; our findings are consistent with these results. Although the causes of renal allograft failure after AP are complex, the aforementioned condition may be an one of underlying causes. This assumption may be supported by the relatively short allograft survival observed in KT recipients here.

Here, we identified the factors contributing to AP in KT patients. The multivariable analysis demonstrated that alcohol consumption, gall stone disease, and past history of pancreatitis were significantly associated with the AP risk after KT. Alcohol consumption and gall stone disease are traditional risk factors for AP in the general population. Our findings provide evidence that supports their causative roles in post-KT AP. In addition, our findings showed that before KT, ESRD patients with a history of pancreatitis were highly likely to be associated with post-KT AP risk (an increase of approximately 9 fold). Furthermore, after KT, recurrent AP risk remained high and became 9.77-fold higher in patients who developed post-KT AP. A study reported that ESRD patients on maintenance dialysis had a significantly higher pancreatitis risk compared with non-ESRD patients [7]; moreover, female patients, biliary stones, liver disease, and receiving peritoneal dialysis (PD) were suggested to be associated with AP in dialysis patients. Taken together, our findings were consistent with those of a previous study, suggesting that AP can occur any time before or after KT in ESRD patients.

Medical professionals should maintain a high index of suspicion when managing KT recipients with a history of AP, in addition to traditional factors that include alcohol consumption and biliary stones. Compared with the general population, post-KT AP has much poorer outcomes in KT patients.

Although the results of this population-based cohort study are robust, some limitations exist that must be addressed. First, medical information regarding obesity, weight, body mass index, APACHE II score, and other biochemical data, all of which may be associated with pancreatitis, cannot be obtained from the NHIRD. To overcome this limitation, proxy variables such as hyperlipidemia and diabetes mellitus were adopted to attenuate the potential confounding effects. Second, noncompliance of patients with medications can occur to some degree in real-world practice. Prescription drugs related to ISs were analyzed as far as possible; however, information such as patient compliance with ISs could not be determined in this retrospective cohort study. For example, poor compliance with ISs was noted, which may have introduced a bias in the AP-related results; this bias may have remained even after adjustment for the associated confounders. Despite our meticulous and comprehensive study design, data derived from our retrospective case–control study are less evident from a randomized control study because some unknown confounders may have existed and biased the results. Perhaps, a randomized control is considered to validate the association between ISs and pancreatitis risk. Third, AP severity could not be measured through the coding system even though the enrolling criteria of the study were highly conservative. In this study, only cases of severe pancreatitis may have been selected and those of relatively mild AP may have been missed, thereby leading to underestimated results.

In conclusion, KT recipients are significantly associated with AP and adversely with poorer long-term outcomes. Relevant medical professionals should maintain a high index of suspicion in routine practice.

## Supporting information

S1 Table HR of acute pancreatitis in association with sex, age, and comorbidities among kidney transplantation patients in univariable and multivariable cox regression models. (DOCX)

S2 Table Comparison of incidence and hazard ratio of pancreatitis between patients with and without kidney transplantation. (DOCX)

S1 Checklist RECORD checklist. (DOCX)
