# Supplementary material for: Acute pancreatitis risk after kidney transplantation: Propensity score matching analysis of a national cohort
Source: PLoS One. 2019 Sep 11;14(9):e0222169. doi: 10.1371/journal.pone.0222169 (PMC6738600; doi:10.1371/journal.pone.0222169)
Supplement: S1 Table — (DOCX) [file pone.0222169.s001.docx]

S1 Table. HR of Acute Pancreatitis in Association with Sex, Age, and Comorbidities among kidney transplantation patients in Univariable and Multivariable Cox Regression Models.

|  | **Crude** | | **Adjusted^†^** | |
| --- | --- | --- | --- | --- |
| **Variable** | **HR** | **(95% CI)** | **HR** | **(95% CI)** |
| **Gender (women vs men)** | 0.86 | (0.50, 1.46) |  |  |
| **Age, years** | 1.02 | (1.00, 1.05) |  |  |
| **Comorbidity** |  |  |  |  |
| Alcohol-related illness | 5.65 | (2.03, 15.7)*** | 3.85 | (1.36, 10.9)* |
| Gall stone | 6.79 | (3.06, 15.1)*** | 3.43 | (1.45, 8.14)** |
| HCV | 2.51 | (1.07, 5.87)* | 1.64 | (0.68, 3.95) |
| HBV | 0.79 | (0.25, 2.55) |  |  |
| CMV | - | - |  |  |
| Diabetes | 2.18 | (1.14, 4.15)* | 1.58 | (0.81, 3.08) |
| Polycystic kidney disease | 1.91 | (0.47, 7.87) |  |  |
| Hyperlipidemia | 1.07 | (0.60, 1.93) |  |  |
| Previous History of pancreatitis | 14.3 | (7.48, 27.3)*** | 9.94 | (4.98, 19.8)*** |
| Peritoneal dialysis | 1.51 | (0.84, 2.71) |  |  |
| Hemodialysis | 0.95 | (0.51, 1.79) |  |  |

Crude HR, relative hazard ratio; Adjusted HR^†^ : multivariable analysis including comorbidities of alcohol-related illness, gall stone, HCV, diabetes, and previous history of pancreatitis;

*p<0.05, **p<0.01, ***p<0.001
