# Supplementary material for: Acute pancreatitis risk after kidney transplantation: Propensity score matching analysis of a national cohort
Source: PLoS One. 2019 Sep 11;14(9):e0222169. doi: 10.1371/journal.pone.0222169 (PMC6738600; doi:10.1371/journal.pone.0222169)
Supplement: S2 Table — (DOCX) [file pone.0222169.s002.docx]

S2 Table. Comparison of incidence and hazard ratio of Pancreatitis between patients with and without kidney transplantation.

|  | **Kidney transplantation** | | | | | |  |  |
| --- | --- | --- | --- | --- | --- | --- | --- | --- |
|  | **No** | | | **Yes** | | |  |  |
|  | **Event** | **PY** | **Rate^#^** | **Event** | **PY** | **Rate^#^** | **Crude HR (95 % CI)** | **Adjusted HR^†^ (95% CI)** |
| **Acute Pancreatitis** | 4 | 35895 | 0.11 | 41 | 34440 | 1.19 | 10.8(3.87, 30.1)*** | 9.77(3.33, 28.7)** |

Rate^#^, incidence rate, per 1,000 person-years; Crude HR, relative hazard ratio; Adjusted HR^†^: multivariable analysis including gender, age, and comorbidity of alcohol-related illness, gall stone, HCV, HBV, CMV, polycystic kidney disease, and hyperlipidemia, and medication of steroid.

**p<0.01, ***p<0.001.
